# From Recoding to Peptides for MHC Class I Immune Display: Enriching Viral Expression, Virus Vulnerability and Virus Evasion

**DOI:** 10.3390/v13071251

**Published:** 2021-06-27

**Authors:** John F. Atkins, Kate M. O’Connor, Pramod R. Bhatt, Gary Loughran

**Affiliations:** 1 Schools of Biochemistry and Microbiology, University College Cork, T12 XF62 Cork, Ireland; 116308383@umail.ucc.ie (K.M.O.); pramodbhat06@gmail.com (P.R.B.); g.loughran@ucc.ie (G.L.); 2 Department of Biology, Institute of Molecular Biology and Biophysics, ETH Zurich, 8093 Zurich, Switzerland

**Keywords:** StopGo, ribosomal frameshifting, stop codon readthrough, codon redefinition, selenocysteine, bet hedging, DRiPs, ribosome structure, cancer

## Abstract

Many viruses, especially RNA viruses, utilize programmed ribosomal frameshifting and/or stop codon readthrough in their expression, and in the decoding of a few a UGA is dynamically redefined to specify selenocysteine. This recoding can effectively increase viral coding capacity and generate a set ratio of products with the same N-terminal domain(s) but different C-terminal domains. Recoding can also be regulatory or generate a product with the non-universal 21st directly encoded amino acid. Selection for translation speed in the expression of many viruses at the expense of fidelity creates host immune defensive opportunities. In contrast to host opportunism, certain viruses, including some persistent viruses, utilize recoding or adventitious frameshifting as part of their strategy to evade an immune response or specific drugs. Several instances of recoding in small intensively studied viruses escaped detection for many years and their identification resolved dilemmas. The fundamental importance of ribosome ratcheting is consistent with the initial strong view of invariant triplet decoding which however did not foresee the possibility of transitory anticodon:codon dissociation. Deep level dynamics and structural understanding of recoding is underway, and a high level structure relevant to the frameshifting required for expression of the SARS CoV-2 genome has just been determined.

## 1. Introduction

Both the meaning of individual codons and the framing of the readout process can be altered by specific information in individual mRNAs or their encoded product. Such translational recoding is dynamic—it is in competition with standard decoding—and it enriches gene expression. It is especially utilized in the decoding of positive sense single-stranded RNA genomes, though also employed by various DNA viruses [1,2,3,4]. One reason for this preferential usage is to expand the coding repertoire of generally (relatively) small genomes—it is also widely used by the diminutive bacterial IS elements—but it also performs various regulatory functions. 

Here to honor the memory of Steve Oroszlan, we review usage of programmed stop codon readthrough, ribosomal frameshifting and other forms of translational recoding in the expression of selected animal viruses. Stop codon readthrough results in a proportion of the product having a C-terminal extension. Switching to an alternative frame, frameshifting, can, depending on the location of the stop codon in the new frame, yield a product shorter than the product of standard decoding (e.g., alphavirus TF protein). Bioinformatic identification of alphavirus-type occurrences is more difficult when a stop codon occurs immediately in the new frame resulting in few, or no, amino acids encoded by the new frame, the consequence is only synthesis of a proportion of product lacking a C-terminal domain. Many cases of viral frameshifting involve the reading frame switch occurring near the end of the coding sequence and yielding a product longer than the product of standard decoding. With orthoretroviruses the existence of the GagPol polyprotein was well known in advance of the discovery of recoding in their expression. However, for some well-studied viruses such as influenza A virus and especially alphavirus, the existence of the coding sequence for the additional protein whose expression involves a recoding event was not appreciated in advance—for alphaviruses, this extended even after the initial discovery. As even existence of the extra ORFs was ‘hidden’ during a long time of intense investigation, use of the term ‘genetic steganography’ seems appropriate [5,6] even though in several cases, the extra ORFs were apparent at an early stage. One example of the latter is in the RNA phage Qβ where the occurrence of in-frame stop codon readthrough was first discovered [7]. Many overlapping genes in eukaryotes are accessed by ribosomal frameshifting. However, many others are accessed by leaky scanning, and the first overlapping sequences discovered in any DNA [8] or RNA [9] viruses also did not involve programmed ribosomal frameshifting in their expression.

Since ribosomes translate 5′ to 3′ and replication of single stranded RNA viruses progresses 3′ to 5′, switching from translation to replication is a key step as ribosomes can block replicases [10]. One of the functions of frameshifting, as proposed a long time ago for certain plant RNA viruses is to facilitate the switch from translation to replication [11], and another is generation of different levels of structural and enzymatic products [6]. Others include appending an extension to a proportion of the viral capsid proteins [12]. A different type of recoding, StopGo (2A), yields the equivalent of two proteolytic products but via a translational phenomenon without the involvement of a stop codon. Relevant to the timing of the earliest examples of recoding considered below is the start, in 1984, of the crucial general availability of synthetic oligonucleotides of predetermined sequence. Together with other aspects of gene readout, recoding studies have since put to rest the old adage: from tapes to shapes—mRNA can be just as shapely as protein and importantly so.

## 2. Pox Virus, Alphavirus and Retrovirus Dynamic Codon Redefinition

When contextual features in particular mRNAs dynamically redefine UGA to specify the non-universal amino acid selenocysteine, the key feature is the identity of the amino acid specified. Selenocysteine is a structural and functional analog of cysteine in which a selenium atom replaces sulfur to confer an enhanced catalytic activity due to a lower side chain pKa and stronger nucleophilicity. Several selenocysteine-containing cellular proteins, selenoproteins, have important cellular functions in antioxidant defense, cell signaling and redox homeostasis, and one, selenoprotein P serves a key selenium transport and storage function. Low levels of reactive oxygen species (ROS)—side products derived from molecular oxygen—play important roles in the maintenance of the immune system, but excess ROS leads to oxidative stress. Such stress can be caused by infection by a number of viruses including human immunodeficiency virus, influenza viruses, hepatitis C virus, herpes simplex virus type 1 and Epstein–Barr virus [13]. Though the mechanisms are diverse, in some cases the host antioxidant defense enzymes, and especially members of the selenoproteome, are targeted [14,15]. Among the selenoproteome are Glutathione peroxidases—they reduce hydrogen peroxides, and glutathione peroxidase 4 reduces lipid peroxides [16,17]. Deficiency of selenium and associated oxidative stress impacts the host’s innate immune response [18,19]. It can also result in viral selection whereby a normally benign or mildly pathogenic virus becomes highly virulent not only in the deficient host but also in hosts with ‘normal’ selenium levels [20]. In contrast, at least two viruses with a long term survival strategy have incorporated a selenoprotein gene, perhaps to modulate for their benefit, the oxidative stress and inflammation they cause [21,22,23]. The pox viruses molluscum contagiosum and fowlpox have acquired cellular glutathione peroxidase 1 and 4 genes, respectively (the former successfully manipulates the host environment as evidenced by the papules it causes that often need at least 6 months, and sometimes years, to resolve). For most selenoprotein mRNAs including those for glutathione peroxidases, the efficiency of redefining UGA to specify selenocysteine is rather low [24,25] (though selective forces can lead to high efficiency as evidenced by coding sequences with more than 45 UGAs effectively specifying selenocysteine [26,27]). As shown by Gladyshev and colleagues, in the relevant fowlpox mRNA, the SECIS structural RNA element 3′ of the selenocysteine-specifying UGA required for codon redefinition, is in the coding sequence, 3′ of the selenocysteine specifying UGA [22], but not in the 3′ UTR where all cellular counterparts are located [22,28]. It can even function 5′ of that UGA [22]. The exceptional location of the fowlpox SECIS may have been influenced by pox viruses, unlike many other DNA viruses, replicating in the cytoplasm [22]. In eukaryotes 3′ SECIS elements and their associated proteins ‘inform’ ribosomes that a UGA(s) far 5′ should specify selenocysteine by at least mainly acting close to, or at the (first) UGA. How is unknown. Since in vitro transcribed mRNA can function to specify selenocysteine, it is clear that splice junction complex proteins located close to the (first) UGA are not essential. However, especially for mRNAs where selenocysteine specification is efficient, it remains possible that they perform some guiding role, or did so at an evolutionary earlier time [29]. A substantial number of proteins, including eIF4A3 [30] are involved in selenocysteine specification, with several having nuclear and/or cytoplasmic roles [31]. To what extent, exclusively cytoplasmic fowlpox glutathione peroxidase 4 mRNA selenocysteine specification merits investigation. Recently a mass spectrometric based approach revealed facultative incorporation of selenocysteine (or selenomethionine) at regulatory sites of key metabolic proteins whose coding sequence lacks the features thought until now to be required for its presence. The mechanism involved is unknown as is its relevance to viral infection and whether any viral encoded proteins contain selenocysteine by the process involved [32].

Dynamically redefining UGA to specify selenocysteine contrasts with what is commonly known as stop codon readthrough where the identity of the universal amino acid specified by UAG or UGA (rarely UAA) is often of secondary or no importance. Instead, the key feature is continued translation by a proportion of ribosomes. Decoding the genomes of Sindbis, Venezuelan equine encephalitis and related alphaviruses, involves readthrough of a UGA stop codon to synthesize the polymerase as a fusion with other nonstructural proteins. A phylogenetically conserved stem loop within the ∼ 150 nt 3’-adjacent to the UGA increases readthrough by up to 10-fold [33]. Mammalian termination involves four mRNA nts being pulled into the A site by eRF1 [34,35]. Possible 3′ mRNA stem loop structure inhibition of this process with reduced termination efficiency may explain its readthrough enhancement. 

**Figure 1:**
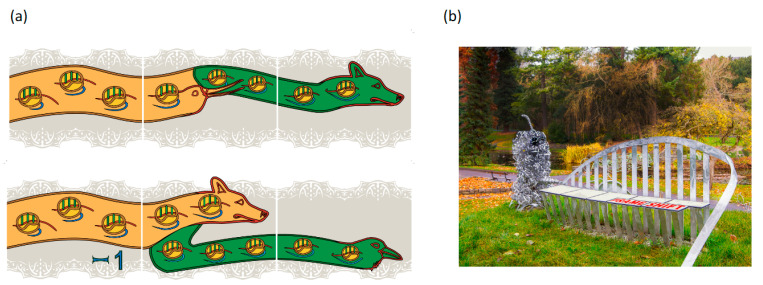
Outreach representations of frameshifting and readthrough. Panel (**a**) depicts retroviral recoding where protease sequencing was important for mechanistic understanding: top, Murine Leukemia Virus gag stop codon Readthrough; bottom, Mouse Mammary Tumor Virus *gag pol* Frameshifting. The head motifs used to depict directionality were inspired by the 1200-year old ‘Book of Kells’ in Trinity College Dublin library. The embedded ribosomes have their A, P, and E sites in green and those shown on the left are at an earlier stage of decoding than those on the right. Correspondingly, on the left the proportion of the mRNA (in red) that has passed through the ribosomes is small in contrast to that shown on the right side, and the nascent peptide emerging from the ribosome (blue) is longer on the right side than on the left. Gag is represented in ochre and Pol in green. These images are from a band of recoding tiles positioned around the middle of the outside walls of ‘a house’ in S.W. Cork, Ireland. Panel (**b**) shows the decoding seat component of the sculpture in the National Botanic Gardens, Dublin entitled ‘What is Life’. The title follows that used by Erwin Schrödinger of the Dublin Institute of Advanced Studies for his 1944 book (and previous year lectures). Both Watson and Crick independently credited the book ‘What is Life?’ as an early source of inspiration for them. A description of the components of the sculpture, including a hammerhead ribozyme and a ribosome can be found at http://whatislife.ie/ (accessed on 1 May 2021). The decoding seat is on a mound overlooking an iconic Charles Jencks 5.5 m high DNA double helix similar to those at Clare College Cambridge and near Cold Spring Harbor Laboratory beach. Each seat panel represents a codon. Three bars below and above each panel reflect its 3nt composition. Starting from the left, or 5′ end, the initial panels reflect all zero frame reading. A proportion of ribosomes shifting to the -1 frame is represented by the first split panel in which part of the panel is offset to the left by one third of a panel length. Continued triplet decoding by frameshifted ribosomes, and by zero frame ribosomes is represented by the panel at the right end.

After studies of adenovirus led to the discovery of splicing in 1977, it was widely assumed, and even stated by a leader of the field in a voluminous monograph, that retroviral GagPol synthesis involved inefficient splicing to generate a subpopulation of RNA with fused gag and pol coding sequences, permitting synthesis of GagPol by standard translation. An in vitro protein synthesis experiment by Philipson, Gesteland and their colleagues with Moloney murine leukemia virus (MuLV) RNA in the presence of yeast amber suppressor tRNA yielded an enhanced ratio of GagPol fusion protein to Gag [36]. Before sequence information was available, this led to the proposal that a single UAG separated *gag* from *pol* which was in-frame with respect to it. However, it failed to dispel the widely held assumption that a splicing event was involved in its natural synthesis. Several of the N-terminal amino acids of the viral protease that cleaves the viral polyprotein, are encoded by the 3′ end of *gag*, and the main, more C-terminal part of it is encoded by the 5′end of *pol* (this viral protease is itself proteolytically released from its GagPol junction position). The key result which definitively showed that MuLV GagPol naturally results from readthrough of the *gag* terminator rather than splicing, was the sequencing of MuLV protease by Steve Oroszlan and his NIH colleagues [37] (Figure 1). They demonstrated that in GagPol the amino acid, glutamine, was present at the position corresponding to UAG in the mRNA. Glutamine tRNA is near-cognate for UAG, Since UAG causes almost 95% of the ribosomes translating gag to terminate, the remaining 5%+ of product, GagPol derives from readthrough of the gag terminator. J. Levin, A. Rein and their colleagues then showed that viral infection does not alter the tRNA(s) that decodes the UAG [38], so the readthrough is mediated by the standard cellular glutamine tRNA. Later an mRNA pseudoknot 3′ of the UAG was shown to be needed to yield significant levels of readthrough [39,40]. More detailed mutagenic analysis, and also structural probing of the pseudoknot and the 8 nt separating it from the UAG followed [40,41,42,43]. Later this pseudoknot became a prototype for studies addressing whether a protonation-dependent switch occurs to induce an active conformation [44]. In addition to the readthrough stimulatory role of the 3′ pseudoknot, Goff, Song and their colleagues discovered that after some GagPol is synthesized, an intriguing secondary stimulator that is also relevant to inhibition of nonsense mediated decay (NMD) comes into play. They identified an interaction of the RNase H domain of the reverse transcriptase component of Pol with the C-terminal domain of peptidyl release factor eRF1. This precludes eRF1 from binding release factor 3, eRF3 [45,46]. This promotes readthrough since delivery of eRF1 via an eRF1/eRF3/GTP ternary complex, is a requisite early step in the termination pathway. The interaction of the RNase H domain with eRF1 is weaker than that of eRF3, but if it is predominately an *in cis* effect that would likely be relevant [46]. This consideration is also pertinent to whether a temporal difference of readthrough efficiency would be expected. Further ribosome profiling experiments [47] could be helpful in this regard. In vivo imaging of one case of recoding, HIV gag-pol frameshifting, suggests that only a small subset of the translating pool undergoes recoding, but that subset does so at a high efficiency [48]. More studies are needed to assess this radical proposal. Whether it is relevant to murine leukemia virus stop codon readthrough is unknown. 

## 3. Retroviral Frameshifting

However, only a minority of retroviruses that express their protease, reverse transcriptase and endonuclease via recoding mediated generation of a GagPol polyprotein do so via stop codon readthrough; most use programmed −1 ribosomal frameshifting as discovered in superb work by Jacks and Varmus [49,50,51,52]. In advance of their initial publication, they (and at the same time one of us, JFA, together with N. Wills), faced a dilemma; how to distinguish between the extended protein arising from standard triplet decoding of a small proportion of the mRNA lacking a specific base due to an earlier slippage event at the polymerase level, and some ribosomal frameshifting on mRNA that had no such deletion (or counterpart addition of two bases)? Eliminating the possible involvement of splicing was simple via mRNA generation in *E. coli* [49], but it was not initially clear to all involved that such expression would not on its own, eliminate the possibility of transcription slippage. With hindsight, though its sequence was unknown at the time, starting with mouse mammary tumor virus (MMTV) sequence would have permitted a simple solution. Its protease gene is in the −1 frame with respect to *gag* and is separate from its *pol* which is in the −1 frame with respect to it. Since two successive −1 frameshifting events are required to generate its GagProPol fusion, the efficiency of the first one is high, 23%, and at a level that RNA heterogeneity could at the time have been distinguished, whereas the much lower level such as occurs with the single exceptional event that required Rous sarcoma virus GagPol synthesis, was problematic. Determination of the relevant nucleotide sequence of MMTV, involvement of ribosomal frameshifting in its expression and its key features were reported in 1987 [50]. For that the results of sequencing the relevant MMTV protease by the Oroszlan group [53], which was made available to Jacks et al. prior to their publication, was important. Their counterpart work with bovine leukemia virus [54] was also significant, but neither had the potential to be as mechanistically incisive as their counterpart work with MuLV readthrough. However, the discovery of −1 programmed frameshifting near the end of the *gag* gene of Rous sarcoma virus, HIV and MMTV [49,50,51,52,55] was completely convincing, with numerous investigations following [6]. 

Frameshifting on the main HIV-1 shift site U_1_ UUU_4_ UUA_7_ yields two different products. One derives from realignment from UUU_4_ UUA_7_ to U_1_UU U_4_UU with Phe Leu encoded by the shift site and the other has the sequence Phe Phe with the second Phe encoded by the −1 frame U_4_UU [51] with similar results being found with heterologous expression of the shift cassettes in *E. coli* [56] and elsewhere. Limitation of acylated tRNA cognate for UUA causes an elevated proportion of product with Phe Phe encoded by the shift site [57,58]. This relevant cognate tRNA is particularly sparse in human cell lines derived from T-lymphocytes, the cells that are targeted by HIV-1. Having alternative modes of frameshifting may compensate for different tRNA levels the virus encounters [59]. Furthermore, several studies also report dynamic frameshifting on the main HIV-1 slip site indicating a non-stochiometric ratio of Gag to Gag-Pol [60,61].

A minor historical footnote for the work of the two groups initially involved, about use of *E. coli* to address the potential alternatives of polymerase slippage and ribosomal frameshifting, is that specific ribosomal frameshifting in *E. coli* does occur on several relevant WT retroviral sequences [56]. That the frameshifting level can be greatly elevated by a single shift site nucleotide substitution [56] is incidental to the use of *E. coli* to distinguish the possibilities. There may be a minor contribution of reverse transcriptase slippage to the generation of GagPol in natural situations [62]. There are parallels elsewhere between flanking nascent RNA transcript stem loop formation acting to stimulate specific RNA-DNA hybrid realignment (transcription slippage) and at the translation level programmed ribosomal frameshifting. However, the study establishing RNA structure dependent polymerase slippage challenged a particular proposal for its use in Ebola virus expression [63]. More than 20 years after the discovery of retroviral frameshifting, a frame switch was identified in expression of *Potyviridae* genomes to allow expression of a previously unknown product. Caution was shown about whether the switch was at the ribosome or RNA polymerase [64]. This was finally resolved in favor of the latter [65,66].

## 4. −1 Frameshifting Shift Sites Leading to Coronavirus Frameshifting

One of the important new ribosomal features discovered by Jacks and Varmus in their studies of retroviral frameshifting was that the site at which the shift occurred allowed slippage by two adjacent tRNAs, and so involving a heptanucleotide ‘slippery’ site [52]. This contrasted with the focus by others up to that time in WT bacteria on slippage by just a single tRNA [67,68,69]. (The issue was not dealt with when frameshift products encoded by DNA phage T7 were initially reported [70] though dealt with later for them [71]. While a single tRNA is involved in earlier reported RNA virus frameshifting [72,73,74,75], the status of its potential biological significance is still unknown.) Unsurprisingly, the initial suggestion was for simultaneous tRNA shifting in the ribosomal A and P sites, but it was not long before the hybrid state mode of ribosome movement and the possibility of realignment occurring during translocation was introduced [56]. Then with sequence from an avian coronavirus, infectious bronchitis virus shown in 1987 by Brierley et al. to require −1 frameshifting for its expression [76], extensive analysis of the ‘slippery’ site revealed much about the latitude and efficiency of anticodon:codon re-pairing to mRNA possibilities [77], with later work characterizing the efficiency in infected cells [78]. However, lesser efficiency elsewhere at hexanucleotide, rather than heptanucleotide, shift sites has also been examined [79].

**Knotting Ventured, Knotting Gained.** Pseudoknots were discovered in 1985 [80], and 5 years later predicted by the same group to extensively act as stimulators for both programmed frameshifting and stop codon readthrough [81]. Meanwhile, a 3′ pseudoknot had been identified by Brierley and colleagues as a stimulator for the frameshifting required for expression of a non-SARS type coronavirus, avian infectious bronchitis virus [82,83]. The pseudoknot involved features an unusually long stem 1 compared to many counterparts elsewhere [84].

**Figure 2:**
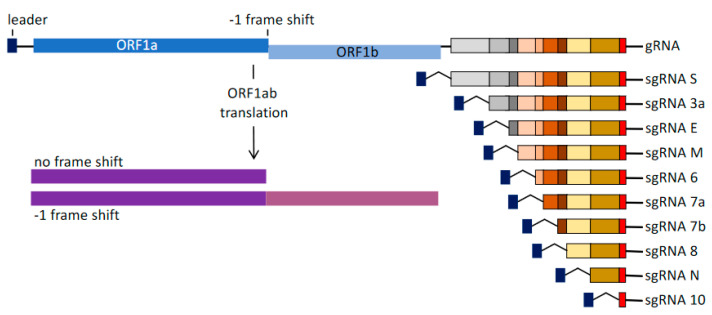
SARS-CoV-2 genomic (gRNA) and subgenomic RNA (sgRNA) structures. −1 FS indicates the site of −1 frameshifting.

Inevitably due to the pandemic that one of the SARS type coronaviruses has caused, most attention has focused on these distinctive types of coronaviruses [85]. While evidence for several long range relevant SARS CoV-2 mRNA structures has recently been obtained [86], the focus here will be on mRNA structures formed wholly by sequence flanking the frameshift site (Figure 2), especially on the 3′ pseudoknot. The 3′ frameshift stimulatory pseudoknot of the SARS viruses (both the first identified SARS, now SARS-1, and that of SARS-2 which only differs by a single nucleotide) has a similar number of nucleotides to that of infectious bronchitis virus. However, early work on the SARS-1 pseudoknot revealed a third stem loop in a region that in infectious bronchitis virus only has loop 2 [87,88,89,90]. Stem loop 3 contains a functionally important RNA dimerization sequence [91].

Small differences in SARS frameshifting efficiency result in different ratios of genomic and subgenomic RNA with implications for optimal virus production [92], Earlier studies with HIV frameshifting also pointed to the potential for beneficial intervention [6,61,93].

Might inhibition of viral frameshifting be part of the host defensive response? The interferon stimulated protein product of C19orf66 inhibits dengue virus replication [94], even though dengue virus is not known to use frameshifting in its expression. It has been reported to interact, and colocalize, with a Zika virus nonstructural protein triggering its degradation via a lysosome-dependent pathway [95], and also to inhibit hepatitis C virus [96]. However, this apparently multifunctional protein has been reported to inhibit the frameshifting involved in HIV expression and in the synthesis of mammalian PEG10, leading to its being named as shiftless [97]. 

**Figure 3:**
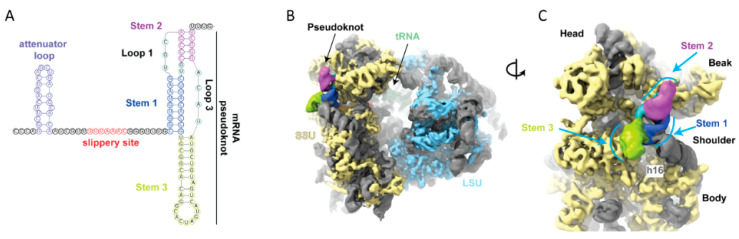
(**A**) Schematic of secondary structure elements regulating -1 frameshifting in SARS-CoV-2. As visualized by cryo-EM [98], the frameshift stimulatory pseudoknot consists of three stems, interconnected by unstructured single-stranded loops. (**B**) Visualization of the stimulatory pseudknot bound to an elongating ribosome at the vicinity of the frameshift site. The intact structured pseudoknot is present at the entry of the mRNA channel on the ribosomal small subunit (SSU). SSU proteins are colored in yellow, LSU proteins in blue, and rRNA in gray. The pseudoknot is colored as per secondary structure description in panel A. (**C**) Pseudoknot as observed from the solvent exposed side of the SSU. Stems 1 and 2 are quasi co-axially stacked, with Stem 3 being perpendicular. Stem 1 interacts with helix 16 of 18s rRNA, potentially aiding the pseudoknot in restricting ribosomal translocation. These figures were adapted from Bhatt et al. [98].

Work led by N. Ban involving several collaborators including us, has revealed the structure of a pre-shift ribosome mRNA complex at an overall resolution of 2.2–7 Angstroms (as well as a later stage ribosome at lower resolution) [98]. In ribosomes paused, one codon before the tandem shift site codons are in the A- and P-sites, the downstream stimulatory 3-stem pseudoknot appears as a bulky structured obstacle at the entrance to the mRNA channel. The pseudoknot is in a corkscrew-like formation, with Stem 3 perpendicular to quasi-coaxially stacked Stem 1 and Stem 2 (Figure 3). Such topology and positioning of the pseudoknot at the ribosome have the potential to resist unwinding by the helicase activity of the ribosome, thereby generating tension on the upstream mRNA relayed up to the decoding center. Wedging of the pseudoknot between the head and body of the small subunit restricts their relative motions. Such motion is very important for translocation, contributing to the ribosomal dynamics that drive frameshifting. It has long been assumed that the fully formed 3′ stimulatory structure presents itself as an obstacle on the ribosome exclusively as the shift-site is over the P- and A-site tRNAs. However, in this cryo-EM structure, the first codon of the slip site was observed in the A-site, suggestive of a progressive slowdown effect. Accordingly, in the cryo-EM structure, one translocation event further than the observed pause state would cause dissolution of the GC-rich base of Stem 1, leading to disruption of the stimulatory pseudoknot, the energy barrier responsible for enhancing the frameshifting event [98]. 

## 5. More Recently Identified Viral Recoding Points to Greater Mechanistic Diversity

Following his Ph.D. in astrophysics, development of a new software program by post-doc Firth, allowed him, along with other members of our lab and collaborators in the 2008–2012 period, to identify recoding utilization by several viruses including influenza A, several mammalian and insect flaviviruses, a second occurrence in arteriviruses, cardioviruses, alphaviruses, *Potyviridae* plant viruses and a novel dicistrovirus ORF (and as considered above 3′ stimulators for alphavirus stop codon readthrough; in addition to overlapping ORFs accessed by leaky scanning, etc.). 

Ribosome profiling is also proving valuable in identifying and characterizing frameshifting and stop codon readthrough [99,100], but will not be considered here.

**Influenza A: +1 frameshifting**: While shifts to the −1 reading frame are well known in virus gene expression, shifts to the other reading frame, by either by +1 or −2 frameshifting, are much less common (though the +1 frameshifting utilized in the decoding of a few chromosomal genes has been intensively studied). However influenza A, a single-stranded, negative-sense, segmented RNA virus, utilizes a low level of +1 frameshifting to express a protein designated PA-X. PA-X has the same N-terminal endonuclease domain as the canonical PA protein which is a subunit of the viral polymerase [101,102]. PA-X is involved in suppressing the adaptive immune response and in host cell shut off [101,103,104,105] for which the key amino acids have been identified [104]. The frameshift site is UCC UUU CGU [101,102]. Though no 3′ recoding signal is known, the A-site codon, CGU is one of the most seldom used codons in mammals and birds. However, while this is relevant for the sparseness of the corresponding aminoacyl tRNA and so for frameshift efficiency, several questions about significance of the identity of the A- and E-site codons remain unresolved [102]. Identification of a frameshift site in influenza virus RNA has pointed to the probable +1 frameshift sites for other viruses including chronic bee paralysis virus and Lake Sinai viruses [102], as well as two new strong, and related, candidates for mammalian cellular gene +1 frameshifting [106]. 

Two approaches have been taken to study the degree of significance of the free energy difference of tRNA pairing in the original, or 0- frame, and in the new frame, i.e., the thermodynamics of frameshifting [107,108]. One of the studies focuses in part on downstream structural stimulator buffering frameshift efficiency with respect to temperature variation [108]. Its results imply that unless relevant unidentified stimulators for influenza A virus frameshifting exist, the frameshifting level should be slightly higher during fever conditions compared to normal body temperature. 

**Flaviviruses**: The Japanese encephalitis virus serogroup of flaviviruses, which includes West Nile virus, utilize −1 frameshifting to synthesize a protein, NS1’ that is important for viral invasiveness [109,110]. NS1’ induces expression of micro RNA-22 (miR-22) which antagonizes host mitochondrial antiviral-signaling protein (MAVS), and so inhibits IFN-I production thereby facilitating viral replication [111]. High levels of NS1’ generating flavivirus frameshifting are dependent on an RNA structural stimulator, a pseudoknot, 3′ of the frameshift site [109,110]. Japanese encephalitis virus infects about 68,000 people per year in the Asia Pacific region with about 20–30% ending in patient death. The live attenuated strain, JEV SA14-14-2 used for vaccination has a synonymous mutation of the pseudoknot that inactivates frameshift stimulation [112,113]. Consequently, after vaccination NS1’ is absent in sera, in contrast to the situation upon infection with most virulent viral strains, thereby creating a distinguishing diagnostic biomarker opportunity [114].

A study of the West Nile virus frameshifting 3′ stimulator used optical tweezers to apply tension to single mRNA molecules, mimicking the tension applied by the ribosome during frameshifting. It led to support for a hypothesis that conformational heterogeneity plays a key role in frameshifting and suggests that transitions between different conformers under tension are linked to efficient frameshifting [115]. 

Several insect flaviviruses also utilize frameshifting in their expression [116].

**Arteriviruses and cardioviruses: Proteins binding 3′ of shift sites** Partly because of their distance from shift sites, until 2010, structural RNAs 3′ of shift sites that stimulated frameshifting were generally thought to exert their effects close to or at, the unwinding site within the ribosome’s mRNA entrance tunnel, rather than transiently blocking entrance from the outside. They were only known to involve RNA. However, the 5′ end of the counterpart frameshift modulatory sequence involved in two subsequently discovered occurrences were several nucleotides further 3′ raising the possibility that they acted at the entrance to the mRNA entrance channel. However, as described next their action requires protein binding and given the currently unknown level of steric occupancy, location inference awaits structural studies. As already demonstrated in several bacterial frameshifting investigations, smFRET studies provide invaluable information about frameshifting (we are fortunate to have participated in one such study led by J. Puglisi [108]). Such information and structural insights from cryoEM are complimentary.

The family *Arteriviridae*, like the *Coronaviridae*, are in the order *Nidovirales*, and includes porcine reproductive and respiratory syndrome virus (PRRSV) which is perhaps the most important pig pathogen. PRRSV, whose genome is ca.15kb, productively utilizes three different frameshift-derived products. Programmed −1 frameshifting occurs just 5′ of the junction of its long ORF1a with its long ORF1b to synthesize its replicase polyprotein [117,118]. However, within the nsp coding sequence of ORF1a, PRRSV also employs 20% efficient −2 frameshifting, to generate a multifunctional product, nsp2TF (Trans-Frame), consisting of the N-terminal of nsp2 and a unique C-terminal domain [119,120]. Immediately 3′ of the shift site, there is a stop codon in the -1 frame. The −1 frameshifting also occurs at the same shift site and generates a product, nsp2N, that is a truncated form of nsp2 [121].

**Figure 4:**
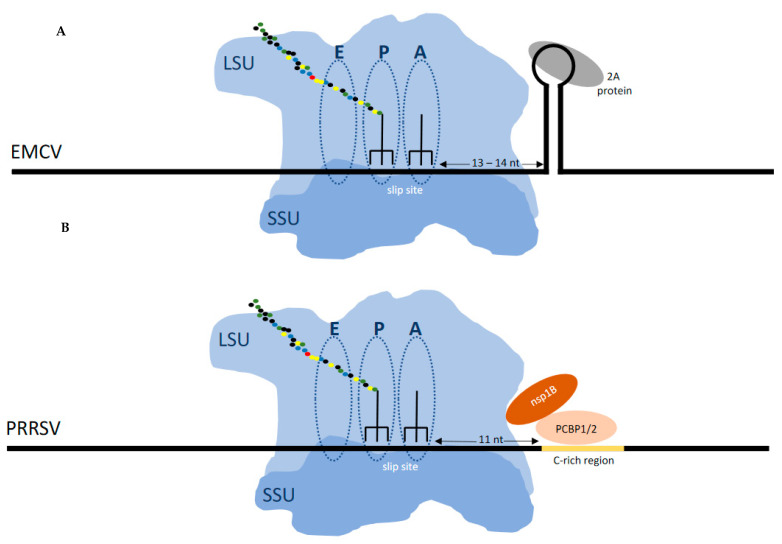
(**A**) Frameshifting in EMCV (and TMEV) is stimulated by a protein:RNA complex positioned at the leading edge of the ribosome when the decoding center is on the shift site. The viral encoded 2A protein, which is encoded 5′ of the shift site, binds to the 3‘ stem loop to stimulate frameshifting. (**B**) Frameshifting in PRRSV is stimulated by a protein:RNA complex that requires dimerization of the virally encoded nsp1B protein and the chromosomally encoded polyC binding protein which bind to the 3‘ stem loop to stimulate frameshifting.

The 3′ stimulator for the frameshifting occurring at that shift site is not an intra-mRNA structure but instead 10nt 3′ of the shift site there is a protein binding site (CCCANCUCC). The viral protein nsp1 together with host poly(C) binding proteins bind at this site generating a RNA:protein complex that is at the leading edge of the ribosome when its decoding center is at the shift site [121,122,123] (Figure 4). A role for the frameshifting products is described below.

In contrast the cardioviruses (*Picornaviridae*) encephalomyocarditis virus and Theiler’s murine encephalomyelitis virus, feature a 3′ stem–loop RNA structure 13 or 14nt 3′ of the shift site. It is to this structure that a viral encoded protein, 2A, which is encoded 5′ of the shift site, binds to cause frameshifting. Early in infection there is no detectable frameshifting, but late in infection with increasing 2A synthesis frameshifting efficiency reaches 70–80% depending on the virus [124,125,126,127]. 

**Alphaviruses:** In addition to viral encoded proteins binding to sequence 3′ of frameshift sites and interacting with the leading edge of the ribosome to link frameshift efficiency to intracellular life cycle stage, other types of viral recoding signals are emerging. Alphavirus frameshifting is providing likely candidates. This frameshifting permits expression of a short −1 frame ORF which is embedded wholly within sequence encoding the overlapping protein [128]. 

The 3′ structural stimulators for the frameshifting utilized by some alphaviruses, e.g., Middelburg virus, are pseudodoknots [128,129]. For Semliki Forest virus despite bioinformatic [128,129] and structural probing [59] evidence for a single stem loop, a relatively short sequence without apparent intra-RNA stem loop potential on its own can mediate a significant proportion of the expected level of frameshifting [129]. How that sequence operates has not yet been reported, but analogous features are known elsewhere. (Single stem loops are known for other animal viruses, e.g., human T-lymphotropic virus type 2 [130].) The frameshift site for essentially all alphaviruses with the exception of Bebaru virus is the same as for HIV-1 and the majority also have G 3′ to the shift site as does HIV-1 [128]. For at least Sindbis virus, as for HIV-1, there are two frameshift products that differ by a single amino acid. Relevant tRNA availability determines the product ratio [59].

Given the efficiency of frameshifting seen from alphavirus frameshift cassettes with little alphavirus sequence 5′ of the shift site, it has come as a surprise that a nascent peptide feature is an important component of alphavirus frameshifting. This will be presented next though the inter-relationship of the nascent peptide influence and the 3′ stimulators has not yet been reported, and there is currently a dilemma regarding comparisons with levels in viral infected cells. The part of the product protein encoded 5′ of the frameshift site can cotranslationally form either of two topologies. The minor topology, which forms 20% of the time, has an extra trans-membrane domain compared to the more abundant topology. It has been reported that the force generated by the translocon-mediated membrane integration of the extra trans-membrane domain within the minor topomer, leads to a tension on the ribosome. In conjunction with the frameshift stimulators 3′ of the shift site [129], this tension has been proposed to be a stimulator for the -1 frameshifting [131] that results in synthesis of the trans-frame encoded virulence factor protein. In other words, mechanical force by translocon-mediated nascent protein insertion into membranes influences frameshifting efficiency [131], likely by modifying anticodon:codon interactions. (For frameshifting involving a particular pairing between mRNA 5′ of the shift site to rRNA [132], stimulation of −1 frameshifting was shown to occur without lengthening of the ribosomal rotated state lifetime, providing evidence that this enhances frameshifting by inducing mechanical force [108].)

Further diversity of viral frameshifting signals associated with different ribosome interactions may await discovery. mRNA:rRNA pairing in the ribosome mRNA exit channel is known to be important for a substantial proportion of functionally utilized bacterial frameshifting occurrences, and suspected for one mammalian chromosomal gene family [6]. In relation to 5′ stimulators acting at the RNA level, unresolved issues relating to 5′ stem loops in both SARS CoV1 and CoV2 mRNAs and elsewhere also merit additional investigation [133,134].

**Dicistroviruses:** Despite the two cistron implication from their *Dicistroviridae* family name, Israeli acute paralysis and related dicistrovirus have a third ORF 3′ of the intergenic IRES that, in the +1 frame, overlaps the 5′ region of ORF2 [135]. In addition to directing ORF2 translation, the IRES also directs translation of the overlapping ORF and does so by forming a UG base pair adjacent to the IRES’ P-site tRNA mimicking domain [136]. The reason to mention this is its relationship to the intriguing situation with a different dicistrovirus, cricket paralysis virus, where recoding may be involved. In that virus, a subset of ribosomes recruited to the IRES bypasses 37 nucleotides downstream to resume translation at the +1-frame 13th codon—it is not AUG [137]. This is different from the translational bypassing known for a bacterial virus and will have much to reveal about decoding versatility. 

## 6. Releasing a Newly Synthesized Peptide without Terminating Decoding: StopGo and Its Use to Determine the Degree of Importance of Retroviral Recoding

For many years it was assumed that foot and mouth disease virus encoded a 2A.2B polypeptide (as part of an L-1ABCD-2A-2BC-3ABCD polypeptide) that was proteolytically cleaved to yield separate 2A and 2B containing proteins. However, Ryan and his colleagues discovered that instead of proteolytic cleavage, a remarkable translational phenomenon, often termed 2A or StopGo, is responsible for generating the separate products [138,139,140,141]. The sequence at which StopGo operates involves adjacent Pro Gly Pro codons and occurs when these codons are in the ribosomal E-, P-, and A-sites, respectively. Despite no stop codon being present, the translating ribosome releases the upstream encoded L-1ABCD-2A polyprotein. Decoding continues with the second proline codon specifying the N-terminal amino acid of the downstream encoded 2B protein. It is separately released after completion of its synthesis. For StopGo to occur, specific amino acids encoded 5′ of the Pro Gly Pro sequence need to be present at particular regions of the ribosomal exit tunnel, with the identity and location of other amino acids influencing StopGo efficiency. These findings have led to the presumption that interaction of that nascent peptide with the exit tunnel caused a ribosomal conformational change which allowed exceptional hydrolysis of the ester linkage of peptidyl-tRNA Gly [138,139,140,141]. In addition to foot and mouth disease virus and other aphthoviruses utilizing StopGo, cardioviruses also do so, though enteroviruses and rhinoviruses do not [142]. However, such unrelated viruses as double stranded *Reoviridae* (e.g., Human rotavirus C) and the insect viruses, iflaviruses and dicistroviruses, also utilize StopGo [142]. Further studies on the degree of importance of StopGo for expression of foot and mouth disease virus [143] and further mutagenesis of its StopGo cassette [144,145] are highly desirable.

As StopGo signals are highly efficient, autonomous and functional in all tested eukaryotic systems, they have already had numerous applications [146]. One example is in dual reporter constructs with a test sequence introduced between two StopGo cassettes, the 5′ of which is fused to the coding sequence for the upstream reporter, and the 3′ one is fused to the downstream reporter coding sequence. This permits assays of both reporters without distortion due to fusion with the test sequence product [147,148]. Another example related to the material above is the use of StopGo to discern the degree of importance for HIV-1 of expressing its Gag and Pol as a GagPol fusion via frameshifting instead of separately. With a mutant that contained the StopGo cassette between gag and pol, only one third of WT infectivity was retained [149]. Apart from packaging reasons, a possible advantage of reverse transcriptase being included in a polyprotein can be inferred from earlier work which pointed to reverse transcriptase in GagPol being inactive prior to sequestion/packaging of the virion RNA [150].

## 7. Implications for Synthetic Manipulation of Frameshifting, Readthrough and StopGo for the Successful Delivery of Nanoparticle-Complexed Nucleoside-Modified RNA and of DNA Vaccines

The vaccine triumphs recently achieved with synthetic structured mRNA containing naturally occurring modified uridines, nanoparticle complexed for delivery, raise interesting mechanistic issues and open up important future opportunities. The modified uridines minimize recognition by RNA binding proteins including endosomal Toll-like receptors that are involved in the innate immunity reaction to foreign RNA as well as increasing its translatability, while the lipid nanoparticle facilitates delivery [151,152,153,154]. (CureVac does not use RNA base modification [155], but does utilize other features such as a histone mRNA stem loop that also stabilizes the mRNA.) Further developments including incorporating alphavirus-derived RNA polymerase encoding ‘self amplifying’ features are extending the capabilities. While the potential of these technologies for vaccines for other viruses (e.g., flaviviruses [156]) and certain forms of cancer is actively being explored, there is likely also potential for specific human genetic disease amelioration with a study on liver repair providing an indicator [157].

Of relevance here is the implications of these technologies, and of adenovirus backbone-based DNA vaccine technology, for independent studies to artificially modulate frameshifting or readthrough efficiencies. 

Some early studies explored whether frameshifting structural stimulators could involve inter-RNA pairing instead of intra-mRNA pairing. Synthetic RNA oligos, several involving modified bases, complementary to sequence 3′ of a shift-prone site functioned as trans-acting components of an active stimulatory structure [158,159,160,161]. In theory this approach could be used to create compensatory frameshifting that might ameliorate the symptoms of genetic disease due to specific frameshift mutants. However, there were no illusions about the impractability at the time due to limitations of oligonucleotide delivery, and that even though major efforts were being made to solve the oligonucleotide delivery issue, this potential use was way down the priority list and not an incentive for the intense delivery efforts undertaken. The new technologies used in the COVID vaccines have the potential, for several genetic diseases, to circumvent consideration of such indirect schemes. However, for at least the initial immune response, short term expression is adequate, whereas for other purposes synthetically delivered RNA with the additional capability of self-replication, is being actively explored. 

Nevertheless, in contrast to viruses such as measles and polio that are disseminated from the initial infection site via lymph or secondarily blood, for mucosae disseminated viruses the length of immunity following an initial infection can be short with consequent implications for herd immunity [162]. 

**Figure 5:**
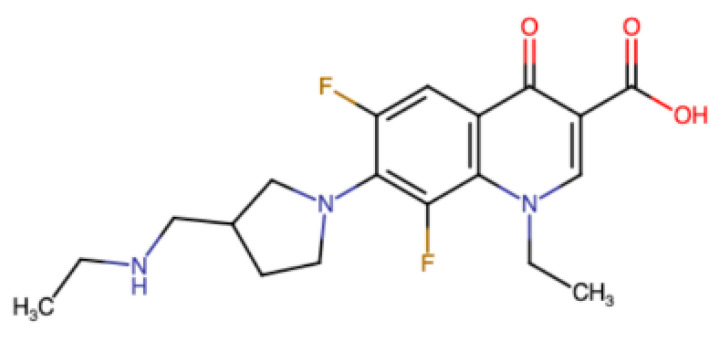
Cartoon structure of merafloxacin [165], an inhibitor of SARS-CoV-2 frameshifting [98,165].

For this and other reasons, the new vaccine technologies do not displace the desirability of identifying good viral targets and, specific for them, cheap stable safe inhibitory drugs. Such compounds have potential benefit not only for those living in areas with few vaccination opportunities or low vaccine uptake. To avoid resistant mutants, a combination of drugs is desirable, and has potential to inhibit a wide variety of even currently unknown SARS-type viruses. Though further development is needed, for the COVID causative virus there have already been numerous reports of protease inhibitors, including [163,164]. The single essential ribosomal frameshifting event required for decoding that virus is also an appealing target and some inhibitors of it have been reported e.g., [98,165]. The SARS CoV-2 frameshifting inhibitory compound studied in those reports is merafloxacin (Figure 5). It inhibits viral propagation in tissue culture cells. 

Is the new RNA vaccine technology relevant to the multiple serotypes of foot and mouth disease virus which, as is widely known, is highly infectious? Due to the cost and time needed for immunological protection it does not detract from the appeal of a drug that would target the form of recoding essential for that virus. As described in the section immediately above, the recoding involved is StopGo. In part as StopGo, at least involving the known sequence characteristics, does not occur in mammalian gene expression, when anti StopGo compounds are identified, they have significant potential to be important antiviral drugs [166].

## 8. Finding Framing Imperfection, or Rather Its ‘Trade-Off’ Occurrence, Despite Crick’s ‘Half-Right’ Reason for Thinking It Would Not Exist

The 70–80% efficiency of cardiovirus frameshifting is quite a contrast to titin synthesis where successful decoding involves the avoidance of frameshifting for >30,000 codons. Actually, for years it was thought that the selective pressure to avoid frameshifting was so great that even frameshifting errors would not be detectable [167,168] and it was not until 1972 that this perception changed. Among a set of mutants induced with a frameshift specific mutagen, the ‘leaky’ ones were discarded as it was thought they could not be ‘real’ frameshift mutants, and the others were considered to be ‘real’ frameshift mutants [169]. However, scepticism about decoding rigidity led to an examination of the ‘real’ frameshift mutants, and identification of small amounts of full-length protein, i.e., they were also ‘leaky but at a lower level [170]. If real frameshift mutants were involved, and compensatory errors at the transcription or translation level allowed synthesis of some full length product, how could it be distinguished at what level the errors occurred? A collaboration with Gorini’s lab, which had isolated single fidelity mutants, permitted the identification of error frameshifting by WT translation components [170]. This followed earlier work on mutants of tRNA and other translation components that perturbed framing and whose identity were later elucidated [171]. Crick was right about a central aspect of readout, translocation, being fundamental as it involves subunit ratcheting [172,173]. However, as we celebrate what is now the 60th anniversary [174] of ‘his’ discovery of the mode of readout [175], 3 years after predicting the existence of the molecule that later became known as tRNA [176], he did not foresee [168] that anticodons could temporarily ‘loose their grip’ of mRNA and so the potential for re-pairing to mRNA in a new frame. Relevant to primordial decoding, translocation can occur at a very low level in the absence of EF-G and GTP due to intrinsic tRNA:ribosomal features [177,178,179]. However, not only is EF-G (and EF2) mediated GTP hydrolysis [180] very important for speed, though its initial binding may contribute to some relevant loss of contacts, in part by stabilizing tRNA:mRNA pairing in the A-site, it is overall hugely significant for restraining unselected frameshifting and enabling selected 3′ frameshift stimulators to function [108,181,182,183]. The extent of importance raises the issues not just of proto-anticodon:codon dissociation and framing in primordial protein synthesis but also of mRNA diffusion. The possibility that one type of recoding stimulator involving mRNA:rRNA pairing may be a remnant of one relevant component is discussed elsewhere [184]. However, while the sophistication of recoding is a natural focus of attention, it needs to be balanced by the long existence of erroneous frameshifting and non-canonical initiation being exploited as two of many facets of the ‘arms race’ between viruses and their hosts.

## 9. Utilizing Imperfections, Including of Framing, to Inhibit Viruses (and Cancer): DRiPS

While the ubiquitin/proteasome pathway generation of viral peptides is important for immunity, a different defensive strategy for obtaining viral peptides to display for antiviral targeting will be considered next. At least for the frame maintenance component of genetic readout, for viruses and cancer cells, the optimal balance between translation speed and accuracy, is likely greatly different than that for normal organismal gene expression, where cellular economy is one of the relevant considerations (synthesis of the extra long protein, titin requires ribosomes maintaining frame for over 30,000 codons). Might the selective advantage for many viruses and cancers of high speed translation have an associated weakness that is exploited defensively? Following viral infection there is an extremely rapid immune system display of peptides derived from viral genes including from those whose products are generally highly stable proteins. At least partial independence from peptides derived from proteins at the end of their normal lifespan, led Yewdell to formulate a hypothesis in which Defective Ribosomal ProductS, DRiPS, that do not achieve functional integration into the proteome, are a major source of antigenic peptides [185]. The peptides are short (ca. 8–11 residues) and are quickly degraded unless presented in the MHC class I cells for TCD8+ immunosurveillance [186,187,188]. Unlike class II [189], the class I immunopeptidome is tightly linked to translation, highly dynamic and sensitive to infection, as well as neoplastic transformation and metabolic perturbation [190,191,192]. 

An illustration of the relevance of frameshifting is provided by a recent study of the effects of treating melanoma cells with interferon-γ ((IFNγ) [193]. IFNγ induces an enzyme involved in the breakdown of tryptophan along the kynurenine pathway and leads to tryptophan starvation. Ribosome profiling and other techniques led to the identification of frameshift-derived products and their origin [193]—‘hungry’ tryptophan codons in the ribosomal A-site leading to peptidyl-tRNA dissociation and re-pairing to mRNA in a new frame. The presentation of aberrant trans-frame peptides was detected, including from patient samples, with implications for immune recognition [193]. Limitation of specific fully functional tRNA is known in various other human diseases [194,195], and there is reduced supply in certain brain cells [196]. In some cases, there is known or suspected relevance to the generation of frameshift-derived peptides. 

Despite this study, to date peptides derived from non-programmed frameshifting and stop codon readthrough are a modest minority of the non-canonical peptides destined for immune display. The repertoire of functionally important cellular proteins synthesized is now known to be significantly greater than apparent even a decade ago only in part due to the extent of utilization of non-AUG codons for initiation then being seriously underestimated [197,198,199,200,201,202,203,204,205,206,207,208,209,210,211].

The cellular response to infection by viruses such as cytomegalovirus (a herpes virus) and influenza viral infections, leads to non-canonical initiation by tRNA^Leu^ on CUG codons [212]. Without viral infection treatment of cells with the inflammatory cytokines, Tumor Necrosis Factor (TNF)-α or type I interferon has the same effect. As a virus is shutting down host protein synthesis, the host response of activating non-canonical decoding, and immune display of derived DRiPs, facilitates curtailment of viral spread via recognition by cytolytic T cells [212].

More generally, as described in several of the publications just cited, stress generates novel products that tend to be unstable and which should not be considered ‘noise’ since their peptides are preferentially displayed on the MHC class I peptidome—i.e., the DRiPs hypothesis has now gained wide support [213], with very recent work further elucidating it [214]. 

Though non-canonical translation is a major source of antigenic peptides, it constitutes a small fraction of total cellular translation, and so selectivity is key; the immunopeptidome poorly reflects either the transcriptome or the proteome [215,216]. Numerous studies have been undertaken to explore the important issues raised, but as also mentioned in the next section below, among them are model system studies [217]. It is exciting that technical advances including in mass spectrometry and analysis of its data, ribosomal profiling and other developments are facilitating furtherance of the conceptual advances, so that important understanding is in prospect.

**Figure 6:**
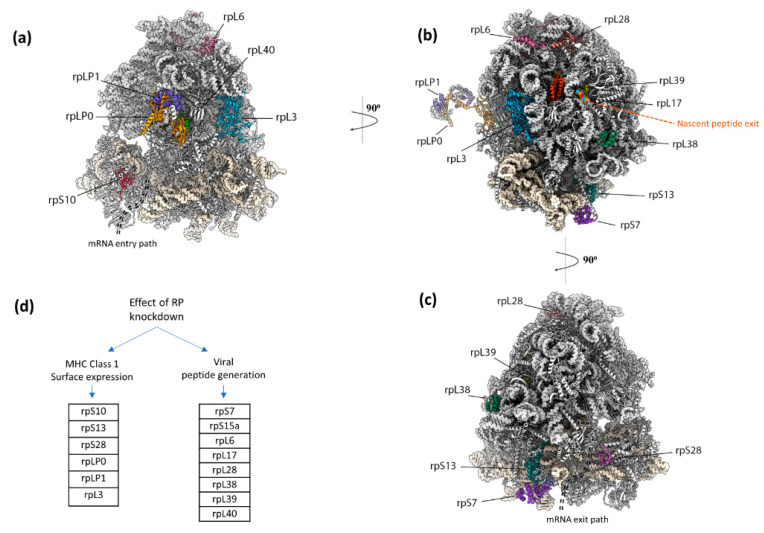
Ribosomal proteins that impact MHC Class 1 peptide generation for immunosurveillance, according to Wei et al. 2019 [192]. (**a**–**c**) Depiction of ribosomal proteins that impact MHC Class 1 peptide generation for immunosurveillance [192]. rpL6 and rpL28, which are adjacent to each other on the large subunit, have opposing effects on viral peptide generation. rpL6 depletion decreases ubiquitin-dependent peptide presentation, whereas rpL28 depletion increases ubiquitin-dependent and -independent peptide presentation. Figure was generated with a human ribosome (PDB 4V6X) in ChimeraX. (**d**) Individual ribosomal protein knockdowns affect immunosurveillance by impacting either MHC Class 1 surface expression or viral peptide generation, and are grouped as reported.

Eukaryotic ribosomes have numerous RNA segments and proteins not required for core protein synthesis as evident from comparison with minimal effectively functioning ribosomes. In addition to ribosome functioning to interact with the translocon being vital for the destination of a subset of newly synthesized proteins, the evolutionary selective pressure for some components may have been related to immune system peptide display, including of DRiPs. Experimental knockdown of expression of each of 80 ribosomal protein genes individually led to the identification of 14 ribosomal protein genes whose products modulate the presentation of specific peptides with at most only minor alteration of the synthesis of the products of standard decoding [192]. The 60S subunit proteins RPL6 and RPL28 which are adjacent on the ribosome and highlighted in Figure 6, perform opposite roles in generating an influenza A virus-encoded peptide. Depleting RPL6 decreases ubiquitin-dependent peptide presentation, whereas depleting RPL28 increases ubiquitin-dependent and -independent peptide presentation. Small ribosomal subunit protein S28 is also highly relevant [192] since its absence increases non-AUG initiation. These findings complement a genome-wide CRISPR screen that identified RPL23 as a negative regulator of CD8+ T cell killing of melanoma cells [218]. Whether there are specialized ‘immunoribosomes’ that perform roles in peptide display distinct from other ribosomes is unclear. (Potential experimental pitfalls relevant to resolving this general topic have been described [219].) 

## 10. Herpes Virus: DNA Viruses Can Use Adventitious Frameshifting to Evade Drugs and Programmed Frameshifting to Perhaps Facilitate Avoidance of Immune Detection

**Drug evasion**: While ribosomal frameshift utilization is well known for bacterial DNA viruses such as T7, λ and *Listeria* phage PSA, the relatively few eukaryotic DNA viruses known to avail of frameshifting is balanced by its importance. For many years the only known relevance of any type of frameshifting to herpes virus gene expression was as a result of D Coen’s lab in-depth study of utilization of non-programmed ‘error’ frameshifting that permits specific thymidine kinase mutants to become resistant to acyclovir and related drugs that are more orally available. These mutants are a serious problem for immunocompromised patients, with many patients worldwide becoming blind. Commonly the mutations are indels in homopolymeric stretches of guanines or cytosines that despite altering the reading frame, do not obliterate thymidine kinase activity. The most common mutant characterized has a single G inserted in a run of 7Gs with net +1 ribosomal frameshifting yielding ∼0.1% of WT activity [220] which is sufficient to permit reactivation from latency [221], and expression of an epitope recognized by T cells [222]. However, it is not enough to activate acyclovir and confer drug resistance. 

**Discovery of a new type of 3′ stimulator**: Another mutation, which is present in 5–10% of clinical isolates, has one cytosine deleted from a run of six cytosines. Though this sequence was suspected to cause frameshifting, an early experiment with the then available dual luciferase assay system [223], yielded no detectable frameshifting. Though later shown to be unrepresentative of the natural situation, it is noteworthy as it illustrates that all such fused dual reporters are incapable of detecting a subset of at least frameshifting and can be misleading. In reality, even though it appears that the frameshifting only involves a single tRNA re-pairing to mRNA in the new frame, the real efficiency of frameshifting is 3–5% due to a 100-fold effect of a then novel stimulator [224]. Due to the absence of downstream stop codons in the sequence brought into the zero frame by the frameshift mutation, ribosomes that do not undergo frameshifting continue translating to the 3′ polyA-tail, i.e., the frameshift mutation creates a non-stop mRNA. The model proposed was that ribosomes stall when reaching the poly(A) tail causing reduced movement of trailing ribosomes. This leads to enhanced frameshifting on the shift site but with subsequent reduced synthesis of the frameshift derived product [224]. The results of several experiments were consistent with this model, and despite the level of net −1 frameshifting, the amount of frameshifting-derived thymidine kinase activity was again 0.1% of WT activity [224]; the same level as for that from the +G mutant. Stalling at Poly(A) is well known [225,226], and there are now other studies on enhanced frameshifting by a trailing ribosome that collides with a stalled ribosome [227], and paused elongating ribosomes can even cause a queue of ribosomes such that initiation can occur at a weak start site [211]. The obvious thought is to regard the non-stop stimulation of frameshifting discovered in the mutant herpes thymidine kinase coding sequence as adventitious frameshifting since it only occurs in mutants. However, might there have been selective pressure to have runs of repeat sequences that are prone to frameshift mutations, and no stop codons in at least one alternative frame to permit ribosomes to reach the poly(A) tail? That could permit ribosome pile-up and enhance the possibility of several nonstandard events. There is no evidence for such ‘bet hedging’ in this case, though, for instance [6,228,229], it has been widely considered elsewhere. Finally, when the run of 5 Cs in a non-stop mRNA is replaced by an HIV gag pol frameshift cassette, frameshifting is also enhanced compared to a derivative with an in-frame stop codon, raising questions as to where else such frameshifting may occur [224]. 

## 11. Immune Evasion

The innate immune response provides the first line of defense against infecting viruses and other intruding pathogens. This ancient-origin response is very important for the initial control of infection and allows time for launching an adequate adaptive immune response. It involves the coordinated activation of several transcription factors that are followed by the activation of type I interferons (IFN), their secretion from infected cells and binding to the surface of adjacent cells. This results in activation of a signaling pathway that in turn leads to the expression of interferon stimulated genes whose products counteract infection. However, host development of relevant IFNγ-secreting cells in response to infection by the economically important **arterivirus, PRRSV**, introduced above, is relatively slow [230]. The −2/−1 frameshift products encoded by PRRSV function to suppress the type I IFN response and delay an effective innate immune response [231].

All three nsp2 variants (nsp2, nsp2TF and nsp2N) include an N-terminal papain-like proteinase domain involved in replicase polyprotein cleavage and have deubiquitinase activity. In vitro transfection experiments showed that their deubiquitinase activity suppresses the innate immune response [232,233,234]. Nsp2TF has been directly shown to function as a deubiquitinase that antagonizes ubiquitination of host cell proteins [120,231]. However, in infected cells nsp2 localizes to the virus replication complex for polyprotein processing. Nsp2TF localizes to the endoplasmic-reticulum–golgi intermediate compartment secretory pathway to stabilize the major structural protein plus facilitate the virus assembly, and it is difficult to determine the location of nsp2N since it contains no amino acid sequence not in nsp2 [119,121,231,235]. In part due to the different locations, the exact mechanism of host innate immune suppression remains to be elucidated. 

Furthermore, frameshifting can also be relevant to avoidance of the adaptive immune response. While many cancers develop immune evasion by inhibiting MHC class I expression in a rather brief period, selection over a long time has refined viral strategies for the same end result and may be especially significant for persistent viruses such as the arterivirus, PRRS and herpes viruses. With respect to PRRS, swine MHC class I molecules are essential components of antigen presentation for the subsequent activation of CD8+ T cells, as well as for the detection of virally infected cells by cytotoxic T cells [236]. The C-terminal region of the TF-domain of the −2 frameshift derived Nsp2TF reduces expression of these molecules in alveolar macrophages, PK15-CD163 cells and monocyte-derived dendritic cells [237]. Nsp2TF promotes arterivirus assembly by interacting with the major viral envelope proteins during their transport along the exocytic pathway and antagonizing host defensive ubiquitination-dependent proteasomal degradation [120]. While this is just one of the ubiquitin-related strategies viruses have evolved [238], unlike the many viral encoded deubiquinases that act on relevant cellular substrates, Nsp2TF acts on other viral encoded proteins [120]. Nsp2TF is targeted to the same compartments of the secretory pathway (ERGIC and Golgi), in which the two major envelope proteins of PRRSV, GP5 and M, accumulate.

Persistence of herpes viruses is more widely known and aspects of two long diverged oncogenic viruses, the gammaherpesviruses, Kaposi’s sarcoma-associated **herpesvirus (KSHV)** and Epstein–Barr virus (EBV), will be considered. These viruses have a short productive phase and a predominant latent infection though expression of a limited number of proteins some of which perform an immunomodulatory role to prevent recognition by the host immunosurveillance system. The most prominent of these is the major latency-associated nuclear antigen 1, that for KSHV is known as LANA1, and its EBV counterpart is EBNA1. Each has a central domain that features repeat sequence. EBNA1 and LANA1 repeat sequences function in part to inhibit major histocompatibility complex (MHC) peptide presentation [239,240,241], and for this specific peptide sequences within the repeat have been identified [242].

However, in 2014, the Chang and Moore group discovered that decoding of the ORFs encoding nuclear antigens of the long diverged tumor viruses, Kaposi’s sarcoma-associated herpesvirus (KSHV) and Epstein–Barr virus (EBV), LANA1 and EBNA1, respectively, is associated with efficient +1/−2 frameshifting [5]. LANA1 and EBNA1 are multifunctional proteins involved in episome maintenance, latency, regulation of transcription, cell cycle, and immune surveillance. The frameshift derived proteins, derive in part from decoding alternative frames in these viral repeat regions [5]. At the nucleotide level their repeat sequences are nearly identical to each other, but their framing is offset so that the zero frame products are quite different (EBNA1′s is glycine-alanine rich). The frameshift derived product from the LANA1 coding sequence has a serine/arginine-rich repeat sequence protein [5]. Similar sequences are found in some of the neurodegenerative associated proteins derived from frameshifting at repeat sequences [196,243,244,245].

**Figure 7:**
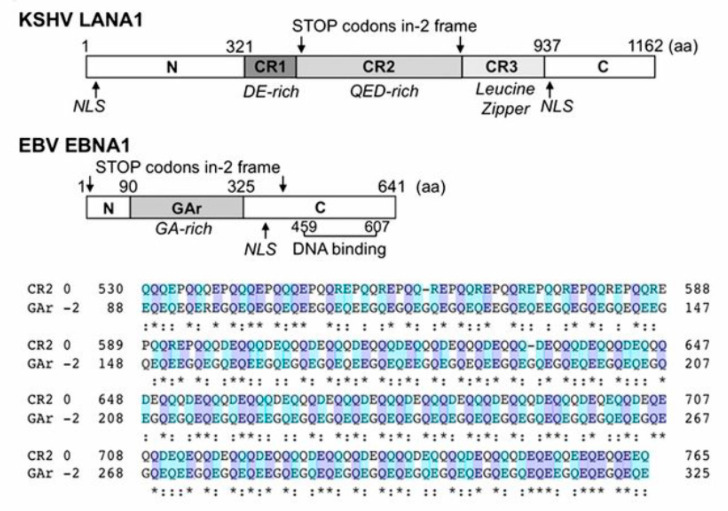
Map of KSHV LANA1 and EBV EBNA1 and amino acid sequence alignment comparing LANA1.CR2 and EBNA1.GAr in the −2 frame (EBNA1ARF). EBNA1 comprises the N terminus, GA-rich central domain, and C-terminal DNA binding domain. Stop codons of the −2 frame are indicated with arrows. Although the EBNA1 GAr (0 frame) has no amino acid similarity to the LANA1 CR, EBNA1ARF has ∼35% similarity to the 0 frame of the LANA1 CR2 domain, and both sequences contain highly acidic QE-rich repeats. From Ref. [5].

The frameshift-derived product from the EBNA1 coding sequence is LANA1-like in having a glutamine and glutamic-rich sequence, implicating a crucial role for these sequences in both viruses (Figure 7). EBNA1 and LANA1 repeat sequences function in part to inhibit major histocompatibility complex (MHC) peptide presentation [239,240,241], with specific peptide sequences within the repeat being identified [242]. Within the mRNA repeat region there are **G-quadruplex** structures whose destabilization increases translation and antigen presentation [246,247] and to which nucleolin binds [248], with consequences for antigen presentation [248,249]. In addition to the well known productive utilization of polymerase slippage at specific linear sequences, for instance by paramyxoviruses, G-rich structures can cause polymerase slippage [250]. While this has not been studied for EBV and KSHV, it has potential to also contribute to decoding of parts of their repeat sequence in what would in WT be an alternative reading frame. Much remains to be elucidated and, only partly because of the nuclear dimension involved, it is challenging.

## 12. Perspective

Animal viruses have impressively exploited selection to beneficially manipulate even the readout within internal coding regions of their own mRNAs. Though occurring without foresight, the level of sophistication gives the appearance of wiliness. What types of unknown animal viruses might feature the most extreme examples of recoding in their expression? At our current stage of ignorance, the best guess would seem to be *Drosophila* neuronal viruses given exceptional readthrough occurring in those cells [251,252,253]. Stretching the definition to include ciliate viruses, then whether *Condylostoma* has been successful in restricting its infecting viruses to those that encode long polyproteins by the expedient of reassigning UAG, UAA and UGA to be sense codons and only recoding them to specify termination by proximity to Poly-A tails [254,255] is of interest (also see [256,257]). Further, features of putative single stranded RNA viruses for the ciliate *Euplotes*, where frameshifting is rampant in chromosomal gene expression [258], are also pertinent. While further understanding of these cases is of great interest, the discovery of new “unknown unknowns” would be even more exciting. At the other extreme, there is no hint of recoding in adenovirus expression (Ulf Pettersson, pers comm, 2021). Perhaps this is not surprising since it generates over 900 alternatively spliced transcripts [259]. Fortunately, when mapping of its protein coding genes was initiated, splicing was an ‘unknown unknown’ and its complexity in that case did not inhibit an approach taken to map its protein coding genes [260].

Finally, in advance of the next Olympic games, perhaps the title of an earlier review “Degree of Difficulty 9.5, Style 10.0” [261] reflects what we know so far about recoding—even though much of even this type of decoding versatility remains to be discovered.
